# Consistent Performance Differences between Children and Adults Despite Manipulation of Cue-Target Variables

**DOI:** 10.3389/fpsyg.2017.01304

**Published:** 2017-08-03

**Authors:** Jessie-Raye Bauer, Joel E. Martinez, Mary Abbe Roe, Jessica A. Church

**Affiliations:** ^1^Department of Psychology, The University of Texas at Austin, Austin TX, United States; ^2^Department of Psychology, Princeton University, Princeton NJ, United States

**Keywords:** preparatory attention, cue-target paradigm, developmental psychology, middle childhood, adolescence, working memory

## Abstract

Two behavioral experiments assessed the plasticity and short-term improvement of task switching in 215 children and adults. Specifically, we studied manipulations of cued attention to different features of a target stimulus as a way to assess the development of cognitive flexibility. Each experiment had multiple levels of difficulty via manipulation of number of cued features (2–4) and number of response options (2 or 4). Working memory demand was manipulated across the two experiments. Impact of memory demand and task level manipulations on task accuracy and response times were measured. There were three overall goals: First, these task manipulations (number of cued features, response choices, and working memory load) were tested to assess the stability of group differences in performance between children ages 6–16 years and adults 18–27 years, with the goal of reducing age group differences. Second, age-related transitions to adult-level performance were examined within subgroups of the child sample. Third, short-term improvement from the beginning to the end of the study session was measured to probe whether children can improve with task experience. Attempts to use task manipulations to reduce age differences in cued task switching performance were unsuccessful: children performed consistently worse and were more susceptible to task manipulations than adults. However, across both studies, adult-like performance was observed around mid-adolescence, by ages 13-16 years. Certain task manipulations, especially increasing number of response options when working memory demand was low, produced differences from adults even in the oldest children. Interestingly, there was similar performance improvement with practice for both child and adult groups. The higher memory demand version of the task (Experiment 2) prompted greater short-term improvement in accuracy and response times than the lower memory demand version (Experiment 1). These results reveal stable differences in cued switching performance over development, but also relative flexibility within a given individual over time.

## Introduction

Executive functions (EFs) have long been highlighted as critical components of child cognitive development. EFs also play a significant role in social development, and have been shown to predict academic achievement (Bull and Scerif, 2001; Espy, 2004; Blair and Razza, 2007) as well as predict long-term health and well-being outcomes in adulthood (Duckworth and Seligman, 2005; Blair and Diamond, 2008). Given the importance of EFs, it is critical that we understand their developmental timeline and task factors that influence performance.

Executive functioning is generally thought to be composed of three core components, updating, inhibition, and cognitive flexibility (Miyake et al., 2000; Diamond, 2006, 2013; Engelhardt et al., 2016). Cognitive flexibility, the ability to adjust to new tasks and demands, emerges relatively late in development relative to other executive processes such as inhibitory control and working memory (Cepeda et al., 2001; Rueda et al., 2005; Luna et al., 2010; Chevalier et al., 2013; Diamond, 2013; Holt and Deák, 2015). The literature on the intersection of cognitive flexibility and working memory is vast and most notably studied using task-switching paradigms (Allport et al., 1994; Rogers and Monsell, 1995; Meiran, 1996; Braver et al., 2003; Kiesel et al., 2010; Koch and Brass, 2013; Forrest et al., 2014). These experimental designs require participants to flexibly attend to discrete task features and to adopt a unique task-set (i.e., load the cognitive and motor processes necessary to respond successfully in a given context) on each trial (Logan and Gordon, 2001).

Adult participants can easily switch between multiple cued tasks (e.g., sorting a target by its color or its shape) on a trial-by-trial basis (Rogers and Monsell, 1995; Meiran, 1996; Monsell, 2003; Kiesel et al., 2010). Other cued switching methods have included categorizing words or images, comparing values of digits, or sorting by direction (up/down vs. right/left) (Rueda et al., 2004; Gade and Koch, 2007; Bunge and Wendelken, 2009; Braverman and Meiran, 2010). There is a performance cost to switching between tasks as opposed to repeating the same task (“switch costs”), and this can be manipulated by stimulus design and cue-target timing (Monsell, 2003). Increased switch costs are frequently observed in children and the elderly relative to young adults (Kray and Lindenberger, 2000; Cepeda et al., 2001; Crone et al., 2006a; Kray et al., 2008; Lucenet et al., 2014; Whitson et al., 2014). There is also brain activity related to loading a trial’s task set (i.e., during the cue period when the trial’s task is indicated but no target stimulus has yet appeared) that is separate and distinct from brain activity related to processing and responding to the target itself (Church et al., 2017).

A widely-used computer based paradigm measuring cognitive flexibility in young children is the Dimensional Change Card Sort (DCCS) (Zelazo et al., 1996, 2013). In this task, children are prompted by a cue indicating how to sort cards on the screen following one rule (e.g., color) for several trials. Then, they are cued to use a different rule (e.g., shape) to sort the same cards for the next block of trials. Children ages 2–4 years have difficulty flexibly updating the new cued task and show decrements in both accuracy and response time when they are cued to use a second rule (Zelazo et al., 2003; Benson et al., 2013). For older children (ages 6 years and up), and adults, the Wisconsin Card Sorting Test (WCST) is used worldwide as a clinical measure of brain injury and frontal dysfunction (Berg, 1948; Grant and Berg, 1948; Romine et al., 2004). In this test, patients are not told how to sort a pile of cards, but are given feedback as to whether each of their card placements is correct. After a certain number of trials, the sorting rules change, and the patient must adjust the sorting strategy in the face of changing feedback. Younger children, and those with frontal lobe injury, are more likely to perseverate on a card sorting strategy and less likely to adjust their strategy in the face of negative feedback. Child performance continues to differ from adults on the WCST well into adolescence (Paniak et al., 1996; Lin et al., 2000).

Previous studies suggest that adult levels of switching performance can be attained by around 12 years of age (Cepeda et al., 2001; Bunge et al., 2002; Luna et al., 2004; Bunge and Wright, 2007), while other work has demonstrated these skills reach mature levels later, around 13-17 years of age (Paniak et al., 1996; Crone et al., 2006b; Davidson et al., 2006). Although developmental trajectories of certain experimental aspects have been explicated (Reimers and Maylor, 2005; Crone et al., 2008), performance interactions with task difficulty and practice over development remain less explored.

There are several proposed accounts for the source of increased performance costs in children. There is evidence of task-set carry-over effects, or task-set inertia (Rogers and Monsell, 1995; Crone et al., 2006a), perseveration errors (Zelazo et al., 2004), and also the inability to disengage attention (Kirkham et al., 2003). A particularly promising framework suggests that the failure to update and maintain rules or task-sets in working memory accounts for performance differences between adults and children (Baddeley and Hitch, 1974; Hofmann et al., 2012; Amso et al., 2014).

### Working Memory as a Support for Task Switching

Cognitive control and flexibility rely heavily on working memory (Baddeley et al., 2001; Blackwell et al., 2009; Badre, 2011; Amso et al., 2014), or the ability to temporarily maintain and manipulate information in one’s mind. In task-switching paradigms, working memory serves to store and flexibly address cues and stimulus properties (Amso et al., 2014). Working memory emerges in infancy (Diamond, 1985) and its capacity increases throughout early childhood and adolescence (Crone et al., 2006c; Davidson et al., 2006; Finn et al., 2010). Given the important role of working memory in driving developmental differences in task-switching performance, it is a promising mechanism through which to manipulate task-set control systems in both children and adults towards the goal of reducing developmental performance differences. Working memory load, or the amount of trial-relevant, yet visually inaccessible, information a participant needs to flexibly update and access on a trial-by-trial basis, might moderate the ability to use preparatory task control. Less taxing of working memory (e.g., remembering 1 piece of information vs. 3 pieces of information) via experimental design could potentially free more cognitive resources for children to switch tasks more quickly.

Interestingly, a recent and growing body of work has demonstrated that computerized cognitive training may improve working memory capacity for single tasks in preschoolers (Bergman Nutley et al., 2011; Diamond and Lee, 2011), young children (Karbach and Kray, 2009; Titz and Karbach, 2014; Wass, 2015), and adults (Buschkuehl et al., 2008; Klingberg, 2010; Mackey et al., 2013). However, there is great debate as to whether these task improvements are generalizable across tasks or cognitive domains (Morrison and Chein, 2011; Melby-Lervåg and Hulme, 2013). While we do not contribute to this debate here, these studies suggest that EFs have some degree of plasticity that might be accessible via task design and time on task.

### The Current Study: Two Experiments

A recent neuroimaging study found large performance and brain activity differences between children ages 9–15 years and adults ages 21–30 years during two-task cued switching (Church et al., 2017). That task had trial-by-trial lexical cues and a high working memory demand, which could in part explain the large age differences in performance. We created the behavioral experiments reported here, in part, to investigate whether the previously observed performance gap observed between children and adults could be narrowed through manipulations of task design, or through practice with versions of increasing complexity. Beyond this goal of improving child task performance, we sought to clarify the age range when child and adult performance distinctions blurred. We also sought to understand maturation of speed versus accuracy on cued switching across middle childhood and early-mid adolescence (Lange-Küttner, 2012). Disentangling age-related performance differences could alleviate neuroimaging performance confounds and inform developmental neuroimaging designs (Church et al., 2010; Greene et al., 2016).

We compared cued switching behavior in typically developing children ages 6 to 16 years and young adults ages 18 to 27 years over two experiments. The cued switching paradigm manipulated the number of cued features and the number of possible response choices over a series of 10 levels. The first few levels of both experiments were relatively easier than the lexically cued, high working memory version of the task used by Church et al. (2017), reflecting attempts to boost child performance with non-lexical cues, response choice reminders on screen, and simpler shape stimuli; the later levels (increasing number of cued features and response choices) were designed to challenge and degrade adult performance. We further aimed to investigate whether improvements in cued switching are possible within session, through extended practice with the task and added task demands. Improvement within a session could allow greater performance overlap between children and adults, creating opportunities to study maturational brain signal differences less confounded by performance gaps.

To examine how age gaps in performance may be influenced by the magnitude of working memory demand, we manipulated this factor across two experiments: (Experiment 1) *Lower working memory demand*, such that when the target appeared, the response choices remained on screen so that participants only had to remember the relevant cued feature. (Experiment 2) *Higher working demand*, where the target was displayed in isolation on the screen, generating substantially more working memory demand per trial, which we predicted would hinder performance.

Given that switch costs are indicators of cognitive flexibility, we also investigated whether switch costs differed between children and adults as we increased the number of relevant stimulus features participants switched between from two, to three, to four. We tested whether age interacted with switch cost size. However, this was a secondary goal as our task was modeled after the pacing for an MRI design that allows separate estimates of the cue and target (Ollinger et al., 2001; Geier et al., 2010; Church et al., 2017), our cue period was quite long (2 s), reducing the size of switch costs relative to more tightly packed or block designs (e.g., comparing a block of A/B alternations to a block of all A trials).

We predicted that age of adult-like task performance would differ between Experiments 1 and 2, such that higher working memory demand would delay the age at which performance became adult-like. Specifically, we anticipated that Experiment 1 (lower working memory demand) would evoke adult-like task performance in early adolescence (i.e., 10–12 years), while higher working memory demand (Experiment 2) would evoke adult-like task performance in mid-adolescence (i.e., 14 years). Finally, we expected that children would improve more within-session than adults, as they had more room for improvement, and presumably less familiarity with these types of tasks.

## Materials and Methods

### Participants

#### Experiment 1

The final sample included 60 children aged 6–16 years (*M* = 11.36 years, *SD* = 2.59, 30 female) and 60 young adults aged 18–27 years (*M* = 20.33 years, *SD* = 2.09, 30 female). Children were recruited through schools in the greater Austin area, the Children’s Research Lab database at UT Austin, and external outreach and recruiting events. Young adults were recruited from the University of Texas at Austin through flyers, online postings, and introductory psychology courses for which students received class credit. Children and young adults who were not enrolled in the study for course credit received $10 in compensation. All participants reported to be in good health, were not taking any psychiatric medications, and had normal or corrected-to-normal vision. We had no hypotheses regarding handedness (10 adults and 19 children were left handed). Participants were matched on age and sex in both adult and child age groups in each between-subject experimental condition (0, 20, or 40% congruency; see task details below). All adult participants gave informed consent prior to participation. For all minor participants, verbal and written assent from the child and parental informed consent was obtained prior to all data collection. All aspects of the study were carried out in accordance with the guidelines and approval of the University of Texas at Austin Internal Review Board.

Additional behavioral data were collected from 25 participants who were excluded. One child did not complete level 10, two children did not complete levels 8 and 9, and one child did not complete level 9 (see description of levels below). These children were included in the analysis but are absent from the relevant level comparisons. Twelve adults (3m/9f, *M* = 19.7 years, *SD* = 1.98) and thirteen children (6m/7f, *M* = 9.8 years, *SD* = 2.37) beyond the 120 described above were excluded from the analyses due to computer errors, incomplete data sets, or failure to meet eligibility requirements. Of the 25 excluded participants, 5 participants were excluded from analysis because their performance was more than 2.5 standard deviations from the mean on 4 or more of the 10 task levels for either response time or accuracy.

#### Experiment 2

The final sample included 47 children aged 6–16 years (*M* = 11.22, *SD* = 2.12, 21 female) and 48 young adults aged 18–25 years (*M* = 20.2, *SD* = 1.73, 26 female). Experiment 2 mirrored Experiment 1 in terms of recruitment, consent, compensation, course credit, and health screening procedures. Three adults and seven children were left handed. As before, participants were matched on age and sex in both adult and child age groups in each experimental condition (0, 20, or 40% congruency).

Behavioral data were collected from an additional 19 participants who were excluded. Two children did not complete levels 8 and 9, and three children did not finish level 9. These children were included in the analysis. Twelve adults (7m/5f, *M* = 19.38 years, *SD* = 1.016) and 7 children (2m/5f, *M* = 8.86 years, *SD* = 1.608) were excluded from the analyses due to computer errors, incomplete data sets, or failure to meet eligibility requirements. Of the 19 total removed participants, 4 participants were excluded from analysis because their performance was more than 2.5 standard deviations from the mean on 4 or more levels for either response time or accuracy.

### Task

**FIGURE 1:**
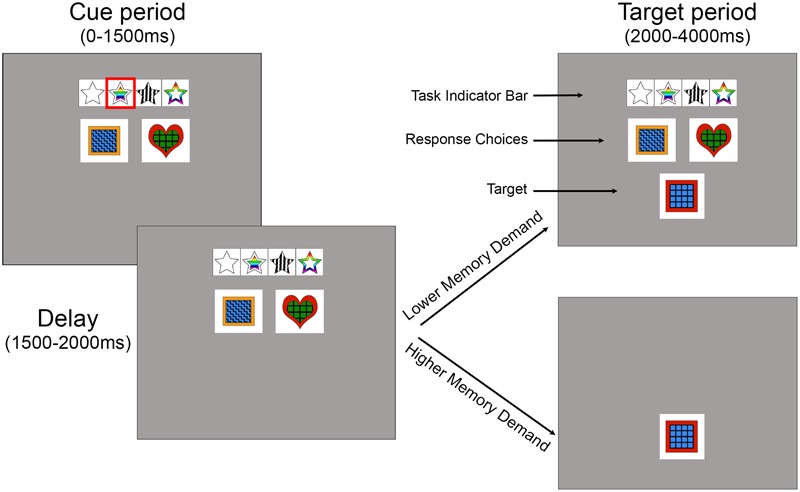
Task structure for Experiments 1 and 2. The cue period lasted from 0 to 1500 ms. During this time, the task indicator bar cued one of 2, 3, or 4 possible features using a red rectangle. The task indicator bar represented the features *shape, inner color, pattern*, and *outer color* (left to right). The response choices for the current trial were indicated in the next row, with a choice for each button (2 in this trial). The delay period lasted from 1500 to 2000 ms. During the target period (2000–4000 ms), participants matched a target to a response choice based on the cued feature via a button press. Experiment 1, the lower memory demand version, is shown in the upper example of the target period, while Experiment 2, the higher memory demand version, is shown in the bottom example (the target is alone on the screen during the target period). The examples above are from a 4 feature, 2-button level (i.e., levels 4–6), and are slightly enlarged relative to the screen size for clarity.

The experiments were created and executed on a 13″ Apple MacBook Pro laptop, using PsychoPy Toolbox (Peirce, 2007) and R Studio (R Development Core Team, 2015). Participants responded by pressing buttons on a hand-held button box connected to the laptop via USB (Delcom Products). On each trial, the participants were cued to attend to one of four stimulus features (shape, inner color, pattern, or outer color) via a red box that highlighted one symbol on a task indicator bar (**Figure 1**). Below the task indicator bar were two or four response choices and subsequently a delayed target that contained a combination of those features. The task was to match one of the response choices to the target based on the cued feature. For example, if the cued feature was “inner color”, the two response choices may have indicated that blue was the left response, and green was the right response. When the target then appeared, if the inner color was blue, a left response was the correct choice (**Figure 1**). Stimuli consisted of red, green, orange, or blue colored squares, hearts, diamonds, or circles created in Adobe Photoshop. The four patterns were zigzag, polka dot, cross, or grid patterns with either red, green, orange, or blue outer color borders. Stimuli measured 4.5 cm × 4.5 cm onscreen.

Each trial included a cue period and a target period. During the cue period, a red box cue appeared for 1500 ms on the task indicator bar that cued which feature the participant would sort the subsequent target by on that trial. The relevant cued feature on any trial was unpredictable. The red box then disappeared and a 500-ms delay occurred before the target appeared for 2000 ms. Each time a target appeared on the screen participants were to match the target to the best response choice (second row) based on the task feature previously highlighted in a red outline in the task indicator row (top row) (see **Figure 1**). No feedback was given on their performance. Each button on the hand-held button box corresponded to a response choice on the screen (leftmost button mapped to the leftmost response choice, etc.). In Experiment 1, the response choices and task indicator bar remained on the screen when the target appeared and throughout the experiment to reduce working memory demand. In Experiment 2, the task indicator bar and response choices disappeared and the target appeared alone to increase working memory demand. The dependent variables were whether the response choice correctly (accuracy) matched the target based on the cued feature, and how quickly the participant made the button press (response time). These responses were recorded and saved through PsychoPy.

The task involved one between-subject and three within-subject primary manipulations. The between-subject manipulation was Congruency: how often the target exactly matched a response choice (0, 20, or 40% of the time). The three within-subject manipulations were (1) Task Switching: if the same task appeared in succession it was labeled a (“repeat”) but if the task changed from the previous trial it was considered a (“switch”), (2) Level: the task became incrementally more difficult by combining and adding different manipulations across nine separate levels or runs (see Level breakdown in **Table 1**), (3) Number of Response Choices: whether a task level had two or four response choices (two or four buttons).

**Table 1:** Layout of level manipulations.

	Within-subject manipulations		
Level	Number of cued features	Number of response choices	Mapping consistency
1	2	2	Consistent
2	2	2	Mixed
3	3	2	Consistent
4	4	2	Consistent
5	4	2	Mixed
6	4	2	Inconsistent
7	4	4	Consistent
8	4	4	Mixed
9	4	4	Inconsistent
10	2	2	Consistent

*Levels varied in difficulty via manipulations of the number of possible cued features (shape, inner color, pattern, or outer color), number of response choices (two or four), and consistency of the response choices mapped on the screen from trial-to-trial. Mapping consistency was “mixed” if the response choices alternated between blocks of the same mappings for several trials, and blocks of different mappings every trial. Consistent mapping indicated that the response choices remained identical for all trials on the level (e.g., blue circle = left response choice, red square = right response choice). Inconsistent mapping indicated that the response choices changed for every trial. Analysis of the mapping manipulation is not reported here.*

### Procedure

Participants were instructed that the task was a matching game during which they were to match a target to the correct response choice based on different features (see **Figure 1**, see **Appendix** file for verbal instructions to participants). Participants were told to respond as quickly and as accurately as possible. Participants completed a practice level of 25 trials for which the stimuli remained on the screen until the participant responded, rather than disappearing after a fixed time limit (self-paced). The practice level shifted between two task features (shape and inner color) and had two response choices (like Level 1). The actual experiment consisted of ten levels. Levels 1, 2, and 10 consisted of 25 trials each; level 3 had 31 trials; and levels 4, 5, 6, 7, 8, and 9 consisted of 53 trials each. Critically, levels 1–6 displayed two response choices while levels 7-9 had four response choices to examine the role that number of output options had on performance. Levels 1 and 10 were identical in format (but with different stimuli) to examine practice effects (**Table 1**). In total, each complete data set for one participant consisted of 424 trials and 25 practice trials and took about 45 min to complete. Due to model complexity and scope of this analysis, we are not reporting effects of Mapping Consistency at this time. All participants were tested individually in a testing room or an empty lab space. The experimental visit lasted approximately one hour, including consenting, study-related questions, instructions, practice, and the experiment.

### Analysis Methods

All analyses were conducted using R Studio (R version 2.0-33, R Development Core Team, 2015). For all of the analyses, we used linear mixed effect models using the “lme4” package (version 1.1.12) in R with maximum likelihood estimation on participants’ average accuracies and median response times. Participants and within-subject variables were used as random effects; only intercepts were allowed to vary for the age transition and session improvement analyses, which required separate models described below. Degrees of freedom calculations are controversial in mixed models due to maximum likelihood estimation^1^ (see “r-sig-mixed-models FAQ”)^2^, therefore to calculate *p*-values, results are reported using Satterthwaite approximations from the “lmerTest” package (R version 2.0-33, Kuznetsova et al., 2016) for degrees of freedom based on the variance-covariance matrices of each model and type III summed squares of factors (Luke, 2016). Non-significant factors were removed from the final model. As such, non-significant results from the full model are reported, as are significant results from the final, pared down model. All comparisons and asymptotic confidence intervals in this study were computed using the “lsmeans” package (version 2.25) in R (Lenth, 2016). Due to the computation time needed for the package to estimate degrees of freedom in complex unbalanced designs, instead of *t*-tests, asymptotic Wald *z* tests are reported that are normally distributed and assume infinite degrees of freedom; they converge with *t*-tests as sample size increases (Luke, 2016). The data and analysis scripts are available at the following Open Science Framework archive^3^.

### Child and Adult Group Performance

In order to determine which conditions led to greater performance similarities between children and adults, we first examined a full model of all the manipulations within each Experiment. The full model included between-subject manipulations of Congruency condition and Age group, and within-subject manipulations of Level (excluding level 10 for analyses not involved in short-term learning) and Task Switching. The models also included the interactions between Age and the other manipulations, as well as the interaction between Task Switching and Levels. Due to the button manipulation (moving from two buttons to four buttons) varying with the levels, we conducted a separate model to specifically examine the role that Number of Response Choices played in task performance.

We then examined the effect of varied working memory demand on performance across experiments by interacting the previously mentioned predictors with Memory Demand in a separate model that combined both experiment’s data sets.

### Transition of Child Performance Across Development

In order to investigate developmental transitions in performance related to task-switch manipulations at a more specific level, we grouped participants into age bins by year of age. The age bins were made by rounding down every participant’s age to the nearest whole number and then creating groups of 6–9 year olds, 10–12 year olds, 13–16 year olds, and adults. The child bins reflect recognition of the multiple maturational factors at play in this age range, and attempt to capture middle childhood, early adolescence, and mid-adolescence. We compared each child age bin’s performance to the adults within each level, using the analysis methods described above. Task Switching was included in the model as a control variable. The sample size in each bin can be seen in **Table 2**. The goal was to detect the age at which child performance was no longer different from adult performance.

**Table 2:** Number of participants (N) in each subgroup for analysis of age transitions within the child group (total age range 6–16 years).

	Experiment 1		Experiment 2	
Year–Age Bin	Mean Age	*N*	Mean Age	*N*
6–9	8 y 6 m	20	8 y 10 m	12
10–12	11 y 5 m	23	11 y 1 m	26
13–16	14 y 8 m	17	14 y 7 m	9
Adult	20 y 4 m	60	20 y 2 m	48

### Short-Term Improvement

To examine on-task learning effects within session, we compared the average performance from both level 1 and level 10, in adults and children, testing for an interaction between Age and Level. These levels were matched on difficulty (two response choices, two cued tasks (shape and inner color), with different stimuli) but differed in the participant’s previous experience with the game.

## Results

We investigated whether manipulating number of cued features, number of response choices, and working memory demand could narrow observed performance differences between children and adults. We describe the effects of these manipulations as Level (1–9), Task Switching (switch costs), Congruency, and Number of Response Choices (2 or 4), and their interaction with Age (Group) below. We report accuracy and response times separately for each experiment: Experiment 1, lower memory demand, and Experiment 2, higher memory demand. We also test across the two experiments for memory demand effects on task performance. **Tables 3–5** summarize the model results.

**Table 3:** Significant predictors and model *R*^2^ in Experiment 1 analyses.

					Model fits			
Analysis	Model step	Fixed effects		Random effects	*R*^2^ Marginal		*R*^2^ Conditional	
		ACC	RT		ACC	RT	ACC	RT
Main	Initial	**Age** Cong **Lvl** **RS** Age:Cong Age:RS **Age:Lvl** **Lvl:RS**	**Age** Cong **Lvl** **RS** Age:Cong Age:RS Age:Lvl **Lvl:RS**	*Slopes:* RS+Lvl *Grouping:* Participant	0.38	0.34	0.82	0.85

Main	Final	**Age** **Lvl** **RS** **Age:Lvl** **Lvl:RS**	**Age** **Lvl** **RS** **Lvl:RS**	*Slopes:* RS+Lvl *Grouping:* Participant	0.38	0.34	0.82	0.85

Number of Response Choices	Final	**Age** **CN** **RS** **Age:CN**	**Age** **CN** RS Age:CN	*Slopes:* CN+RS *Grouping:* Participant	0.47	0.43	0.95	0.94

Age Transitions	Final	**AB** **Lvl** **RS** **AB:Lvl**	**AB** **Lvl** **RS** **AB:Lvl**	*Slopes:* RS+Lvl *Grouping:* Participant	0.58	0.46	0.81	0.84

Within-Session Learning	Final	**Age** **Lvl** Age:Lvl	**Age** **Lvl** Age:Lvl	*Intercept only* *Grouping:* Participant	0.28	0.20	0.65	0.53

*Bolded predictors denote significant fixed effects. ACC, accuracy; RT, response time; Age, Age Group; Lvl, Level; Cong, Congruency proportion; RS, Rule Switch; CN, Number of Response Choices; AB, Age Bins. RS was kept in all analyses except the learning analyses. The intercepts and slopes listed were allowed to vary by *participant*. The marginal *R*^2^ is the variance explained by the fixed effects only, the conditional *R*^2^ includes the full model with random effects (Nakagawa and Schielzeth, 2013).*

**Table 4:** Significant predictors and model *R*^2^ in Experiment 2 analyses.

						Model fits			
Analysis	Model step	Fixed effects		Random effects	*R*^2^ Marginal		*R*^2^ Conditional	
		ACC	RT		ACC	RT	ACC	RT
Main	Initial	**Age** Cong **Lvl** **RS** Age:Cong Age:RS **Age:Lvl** **Lvl:RS**	**Age** Cong **Lvl** RS Age:Cong Age:RS **Age:Lvl** **Lvl:RS**	*Slopes:* RS+Lvl *Grouping:* Participant	0.49	0.37	0.85	0.86

Main	Final	**Age** **Lvl** **RS** **Age:Lvl** **Lvl:RS**	**Age** **Lvl** RS **Age:Lvl** **Lvl:RS**	*Slopes:* RS+Lvl *Grouping:* Participant	0.49	0.35	0.85	0.86

Number of Response Choices	Final	**Age** **CN** **RS** **Age:CN**	**Age** **CN** RS Age:CN	*Slopes:* CN+RS *Grouping:* Participant	0.57	0.46	0.95	0.96

Age Transitions	Final	**AB** **Lvl** **RS** **AB:Lvl**	**AB** **Lvl** RS **AB:Lvl**	*Slopes:* RS+Lvl *Grouping:* Participant	0.54	0.45	0.84	0.86

Within-Session Learning	Final	**Age** **Lvl** Age:Lvl	**Age** **Lvl** Age:Lvl	*Intercept only* *Grouping:* Participant	0.35	0.33	0.62	0.68

*Bolded predictors denote significant fixed effects. ACC, accuracy; RT, response time; Age, Age Group; Lvl, Level; Cong, Congruency proportion; RS, Rule Switch; CN, Number of Response Choices; AB, Age Bins. RS was kept in all analyses except the learning analyses. The intercepts and slopes listed were allowed to vary by *participant*.*

**Table 5:** Significant predictors and *R*^2^ in analyses comparing Experiments 1 and 2.

						Model Fits			
Analysis	Model step	Fixed effects		Random effects	*R*^2^ Marginal		*R*^2^ Conditional	
		ACC	RT		ACC	RT	ACC	RT
Number of Response Choices	Final	**Age** **CN** **Mem** **RS** **Age:CN** Age:Mem **CN:Mem** RS:Mem Age:CN:Mem	**Age** **CN** **Mem** RS Age:CN **Age:Mem** **CN:Mem** RS:Mem Age:CN:Mem	*Slopes:* RS+CN *Grouping:* Participant	0.52	0.56	0.95	0.96
Within-Session Learning	Final	**AG** **Mem** **Lvl** Age:Mem Age:Lvl **Mem:Lvl** Age:Mem:Lvl	**Age** **Mem** **Lvl** **Age:Mem** Age:Lvl **Mem:Lvl** Age:Mem:Lvl	*Intercept only* *Grouping:* Participant	0.32	0.47	0.65	0.73

*Bolded predictors denote significant fixed effects. ACC, accuracy; RT, response time; Age, Age Group; Mem, Memory Demand; Lvl, Level; RS, Rule Switch; CN, Number of Response Choices; AB, Age Bins. RS was kept in all analyses except the learning analyses. The intercepts and slopes listed were allowed to vary by *participant*.*

### Experiment 1: Lower Working Memory Demand

#### Children Were Less Accurate and Slower than Adults

##### Accuracy

We found significant main effects of Age [*F*(1,119.86) = 93.92, *p* < 0.0001], Level [*F*(8,161.13) = 27.29, *p* < 0.0001], and Task Switching [*F*(1,120.33) = 42.01, *p* < 0.0001] on accuracy. Adults were more accurate than children, and repeating a task was more accurate than switching to a new task. Our experimental manipulations from levels 1 to 9 decreased accuracy overall as expected. Contrary to our expectation, there were no significant differences between the Congruency conditions overall [*F*(2,118.03) = 0.29, *p* = 0.75] or in interaction with Age [Age × Congruency: *F*(2,118.03) = 0.21, *p* = 0.81]. Overall performance was unaffected by having 0, 20, or 40% of trials that perfectly matched one response option.

**FIGURE 2:**
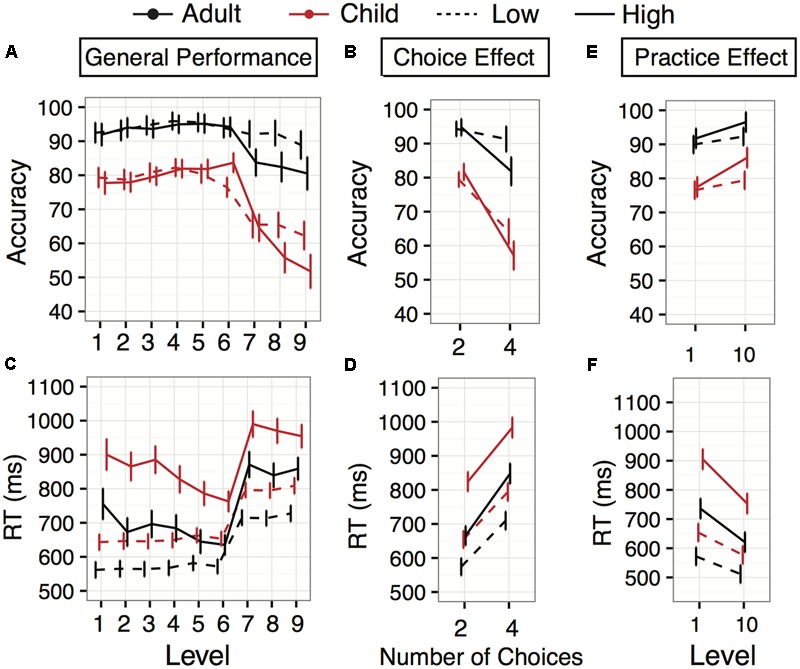
Accuracy and Response times (RT) for Experiments 1 and 2. Experiment 1 (low working memory demand) is displayed with dashed lines, and Experiment 2 (higher working memory demand) with solid lines, for adults (red) and children (blue). **(A)** Accuracy averages plotted across the first 9 game levels for both age groups. **(B)** Accuracy averages across age (same colors as in **A**) separated by number of response choices (2 or 4). **(C)** Response time averages for adults and children across the levels. **(D)** Response time averages separated by number of response choices (2 or 4). **(E,F)** Within-session performance differences: changes in accuracy and response time between level 1 and level 10 (same task parameters as level 1, but performed after 8 intervening, more complex levels). Error bars indicate 95% confidence interval of the mean.

There was an interaction between Age and Level [*F*(8,161.79) = 9.67, *p* < 0.0001]. While adult group accuracy was higher overall than child group accuracy, the greatest gap occurred in the four-choice levels (7, 8, and 9) in which child group performance declined significantly, while adult group accuracy remained relatively stable (**Figure 2A**). The interaction of Age with Number of Response Choices confirmed that the group performance gap was largest in the four-choice levels [**Figure 2B**; *F*(1,120) = 66.06, *p* < 0.0001]. Decreases in accuracy from two- to four-choice levels were greater in children (diff: 15.29%, *z* = 13.03, *p* < 0.0001) than adults (diff: 2.98%, *z* = 2.59, *p* < 0.001).

**FIGURE 3:**
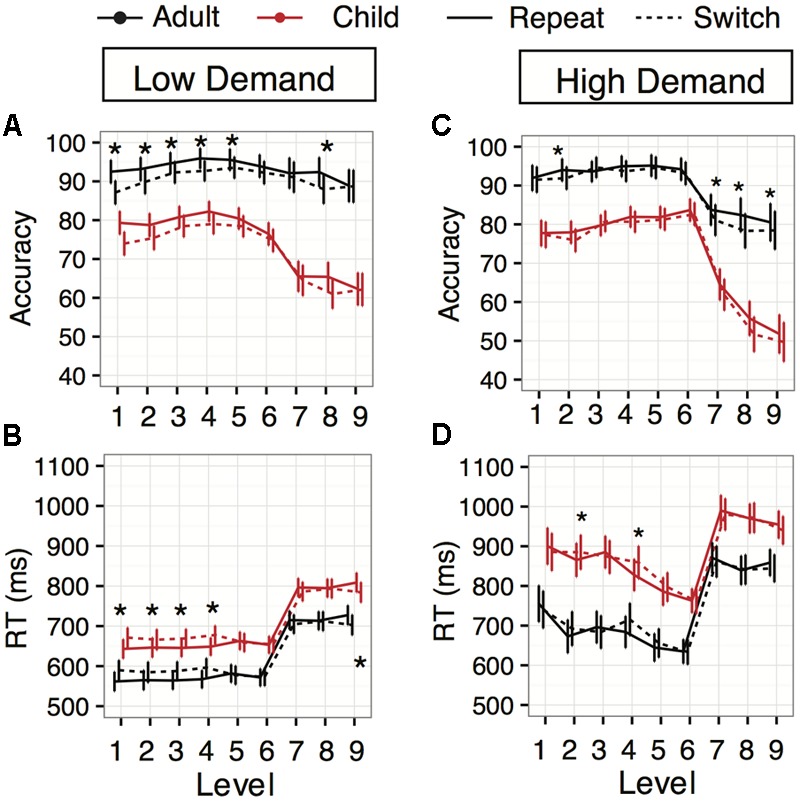
Task-switching performance across levels in Experiments 1 (Low Demand, left panels) and 2 (High Demand, right panels). **(A,C)** Accuracy averages plotted across the levels for repeat (dashed line) and switch (solid line) trials. Adult (blue) and child (red) data are shown. The asterisks denote levels with significant switch costs. **(B,D)** Response time averages for repeat and switch across the levels for adults and children (same color and line contrast). The asterisks above the data represent levels with significant switch costs (slower/less accurate than repeat trials). The Asterisk below the data represents a level with a significant switch benefit. Error bars indicate 95% confidence interval of the mean.

The main effect of Task Switching was qualified by a significant interaction between Switching and Levels (**Figure 3A**; *F*(8,1307.72) = 2.89, *p* = 0.004), with accuracy switch costs appearing in levels 1 (cost: 5.4%), 2 (cost: 3.3%), 3 (cost: 2.3%), 4 (cost: 3.2%), 5 (cost: 2.0%), and 8 (cost: 4.4%) (all *z’s* > 2.04, all *p’s* < 0.05). However, there was no interaction between Age and Task Switching [*F*(1,120.37) = 2.19, *p* = 0.14], suggesting switch costs were similar in both young adult and child groups.

##### Response times

The response time regression analysis revealed significant main effects of Age [*F*(1,120.71) = 31.23, *p* < 0.0001], Level [*F*(8,143.30) = 87.19, *p* < 0.0001], and Task Switching [*F*(1,129) = 5.79, *p* = 0.018]. Adults were faster than children, and repeated cued feature trials were faster than trials that switched (e.g., level 1 repeat: 561 ms, level 1 switch: 589 ms; but see switch cost details below). Our experimental manipulations from levels 1 to 9 increased response times overall. As for accuracy, there were no significant differences between Congruency conditions in overall RT [*F*(2,120.64) = 0.15, *p* = 0.86] or its interaction with Age (Age × Congruency; *F*(2,120.64) = 0.78, *p* = 0.46].

Unlike accuracy, there was no significant interaction between Age and Level [*F*(8,143.63) = 0.59, *p* = 0.78]; children were consistently slower than young adults throughout the game (**Figure 2C**). While there was a significant main effect of Number of Response Choices [*F*(1,120.09) = 638.58, *p* < 0.0001], as can be seen by the slowing in levels 7–9, the four response choice levels (**Figures 2C,D**), there was no interaction with Age [*F*(1,120.89) = 0.23, *p* = 0.63]: the response cost remained constant.

The main effect of Task Switching on response times was qualified by a significant two-way interaction between Task Switching and Level [**Figure 3B**; *F*(8,1184.33) = 8.26, *p <* 0.0001], but not Age [*F*(1,128.82) = 0.027, *p =* 0.869]. Repeat trials were faster than switch trials overall in levels 1 through 4 (*z’s* > 2.87, *p’s* < 0.004), yet repeat trials were equal to switch trials in levels 5 through 8 (*z’s* < 1.48, *p’s* > 0.138) and slower in level 9 (*z* = 3.54, *p* = 0.0004). Thus, we found a loss of switch costs when juggling four tasks, and possibly a small repeat (“stay”) cost when switching among four response choices from options that changed from trial to trial.

#### Transition to Adult-Like Performance Levels Was Observed at 13+ Years

**FIGURE 4:**
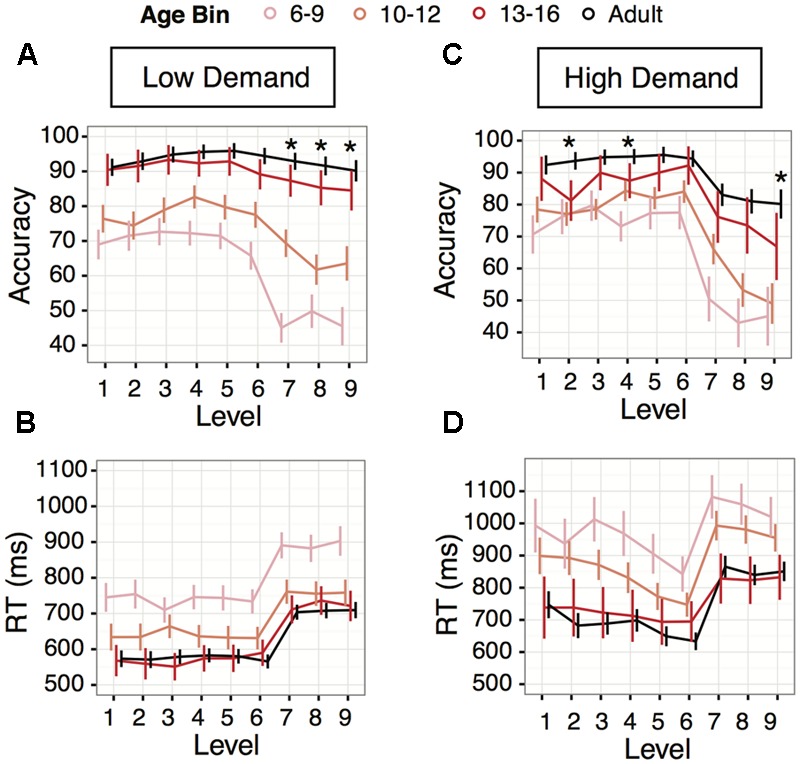
Age transitions in Experiment 1 (Low Demand, left panels) and Experiment 2 (High Demand, right panels). **(A,C)** Accuracy averages by age bins, children ages 6 to 9 years (light pink), children ages 10 to 12 years (orange), children ages 13 to 16 years (red), and adults (blue). **(B,D)** Average response times for the age bins; age increases from top to bottom. For all plots, asterisks represent the levels where the oldest age group (13–16 year olds) are still significantly different from adults. Levels without an asterisk represent levels where the 13–16 year olds’ performance was not significantly different from adults. Error bars indicate 95% confidence interval of the mean.

We binned the children into age groups in order to investigate developmental performance changes in more detail within our sample (ages 6 to 16 years). *Post hoc* paired comparisons within levels revealed that only the 13–16 year age bin had adult-like accuracy performance across levels 1–5 and 9 *(z’s* < 1.70, *p’s* > 0.08*)*; for levels 6–8 all age bins were significantly different in accuracy compared to adults *(z’s >* 2.09, *p’s <* 0.036*)* (**Figure 4A**). For response time, the transition to adult-like response time occurred for all levels in the 13–16 year old children (*z’s* < 1.26, *p’s* > 0.209) (**Figure 4B**). It should be noted that despite adult-like response times in some younger children, even the 13–16 year-olds did not always have adult-like accuracy, so these effects may be driven first by impulsive decision-making strategies in development (Martinez et al., 2017). Improvement in response times then becomes associated with substantially better accuracy starting at around 13 years of age.

#### On-Task Learning within the Testing Period

Both child and adult groups improved their accuracy over the course of the experiment (approximately 45 minutes), shown by a main effect of Level [**Figure 2E**; *F*(1,119.48) = 7.47, *p* = 0.007]. However, the improvement was similar for both age groups, as there was no interaction between Age and Level [*F*(1,119.48) = 0.08, *p* = 0.78], despite higher levels of initial task performance in adults. The same main effect of Level occurred for response time [**Figure 2F**; *F*(1,119.66) = 46.23, *p* < 0.0001], but no interaction with Age [*F*(1,119.66) = 0.651, *p* = 0.421]. Most individual participants improved their response time performance within the experimental session. Overall, 88 of the 119 participants who completed both levels 1 and 10 (68% children; 80% adults) improved in response time. Roughly half the participants, 64 out of 119 (53% of children; 55% of adults), improved in terms of accuracy.

Child accuracy significantly improved by 2.9% (*z* = 2.13, *p* = 0.033), and adult accuracy qualitatively improved by 2.4% (*z* = 2.36, *p* = 0.082). Child response time significantly decreased by 76.77 ms (*z* = 4.76, *p* < 0.0001), and adult response times significantly decreased by 60.55 ms (*z* = 3.78, *p* = 0.0002). Notably, average child group response time (577.0 ms) during level 10 resembled adult group average response time (572.1 ms) in level 1 (*z* = 0.22, *p* = 0.825). This is important given that this adult-like speed is now coupled with greater accuracy relative to initial exposure to the task.

### Experiment 2: Higher Working Memory Demand

#### Higher Working Memory Demand Significantly Affects Performance in Adults and Children

##### Accuracy

The task accuracy regression revealed main effects of Age [*F*(1,95.19) = 82.65, *p <* 0.0001], Level [*F*(8,118.68) = 57.52, *p <* 0.0001], and Task Switching [*F*(1,246.39) = 16.59, *p* < 0.0001]. As in Experiment 1, adults were more accurate, our level manipulations decreased accuracy, and there was a repeat task benefit relative to switching tasks. As in Experiment 1, there were no significant differences between Congruency conditions [*F*(2,96.22) = 0.219, *p =* 0.804] or its interaction with Age [*F*(2,96.22) = 0.786, *p* = 0.46].

There was a significant interaction between Age and Level [*F*(8, 119.04) = 7.835, *p* < 0.0001], despite the fact that with higher working memory demand both child and adult performance declined dramatically in levels with four response choices (levels 7, 8, and 9) (**Figure 2A**). As in Experiment 1, the interaction between Age and Level was driven by a larger performance decline between two to four response choices in children compared to adults [**Figure 2B**; Choice Number × Age: *F*(1,95.03) = 33.95, *p* < 0.0001]. Children’s accuracy differed between the two- and four-response-choice levels by 24.5% (*z* = 18.81, *p* < 0.0001), while adults showed a 13% difference (*z* = 10.03, *p* < 0.0001).

While there was a significant main effect of Task Switching, there was no interaction with Age [*F*(1,251.52) = 0.98, *p =* 0.32]; again, our switch costs were similar over middle childhood and young adulthood. The main effect of Task Switching was qualified by an interaction with Level [*F*(8,1035.96) = 2.09, *p* = 0.034]. The four levels that exhibited significant switch costs were level 2 (cost: 2.08, *z* = 2.03%, *p* = 0.043), level 7 (cost: 2.7%, *z* = 2.58, *p* = 0.009), level 8 (cost: 4.1%, *z* = 3.96, *p* = 0.0001), and level 9 (cost: 2.07%, *z* = 1.96, *p* = 0.049) (**Figure 3C**).

##### Response times

The response time regression revealed significant main effects of Age [*F*(1,95.44) = 40.74, *p* < 0.0001] and Level [*F*(8,117.32) = 138.51, *p* < 0.0001]. There were no significant differences between Congruency conditions [*F*(2,96.88) = 0.29, *p* = 0.75], or their interaction with Age [Age × Congruency: *F*(2,96.88) = 0.51, *p* = 0.60]. Unexpectedly, in Experiment 2 there was no main effect of Task Switching [*F*(1,179.63) = 0.15, *p* = 0.69] or interaction with Age [*F*(1,179.63) = 2.64, *p* = 0.11]. Thus, there were no overall response time-related switch costs in this higher working memory version. However, there was an interaction with Level [*F*(8,941.81) = 2.86, *p* = 0.004], driven by switch costs at levels 2 (cost: 20 ms, *z* = 1.99, *p* = 0.046) and 4 (cost: 32 ms, *z* = 3.23, *p* = 0.001) (**Figure 3D**).

There was a significant interaction between Age and Level [**Figure 3C**; *F*(8,117.29) = 2.12, *p* = 0.041] driven by a larger improvement in response time for adults between level 1 and level 2 (65.2 ms, *z* = –3.84, *p* = 0.0001) than children (17.7 ms, *z* = 1.03, *p* = 0.30) (**Figure 2C**). Still, children were consistently slower than young adults throughout the experiment (*z’s* > 3.98, *p’s* < 0.0002). There was no interaction between Age and Number of Response Choices [*F*(1,95) = 2.22, *p* = 0.14], but there was a main effect of Number of Response Choices [*F*(1,95) = 495.86, *p* < 0.0001], in which four-response-choice trials were 181.9 ms slower for adults [95% CI (160.9, 203.1), *z* = 16.89, *p* < 0.0001] and 159.2 ms slower for children [95% CI (137.8,180.5), *z* = 14.62, *p* < 0.0001; **Figure 2D**].

#### Transition to Adult-Like Performance Levels Is Observed at Age 13+ Years

Following the same procedure as in Experiment 1, we binned the children into age bins to explore performance changes from ages 6 to 16 years. For levels 1, 3, 5, 6, 7, 8, adult-like accuracy was achieved by 13–16 year olds (*z’s* < 1.69, *p’s* > 0.09) (**Figure 4C**). Levels 2, 4, and 9 remained different from adults even in the 13–16 year-old subgroup (*z’s* > 2.27, *p’s* < 0.023).

For response times, the developmental transition observed in Experiment 1 was also seen in Experiment 2: at all levels the 13–16 year olds did not perform significantly differently than adults (*z’s* < 1.74, *p’s* > 0.082) (**Figure 4D**).

#### On-Task Learning within the Testing Period

For accuracy, there was a significant main effect of Level between level 1 and level 10 [*F*(1,95) = 38.68, *p* < 0.0001], and there was a marginal interaction with Age [*F*(1,95) = 3.16, *p* = 0.078], suggesting that, while adults improved from level 1 to level 10 [improvement: 4.8%, 95% CI (1.8,7.7), *z* = 3.15, *p* = 0.0016], children improved slightly more [improvement: 8.6%, 95% CI (5.6, 11.6), *z* = 5.62, *p <* 0.0001; **Figure 2E**]. Overall, 62 participants (68% children; 62% adults) improved in terms of accuracy, and 84 of the 95 participants (87% of children; 89% of adults) improved in response time over the course of the experimental session.

For response times, there was a significant main effect of Level [*F*(1,95.19) = 87.4, *p* < 0.0001], but no interaction with Age [*F*(1,95.19) = 1.66, *p* = 0.201], suggesting that children and adults similarly improved their speed by level 10. Child response time significantly decreased by 152 ms [95% CI (116,188), *z =* 8.40, *p <* 0.0001], and adult response times significantly decreased by 115 ms [95% CI (80,150), *z =* 6.44, *p <* 0.0001] (**Figure 2F**). Similar to Experiment 1, the child group average response time during level 10 was not significantly different from the adult group average response time during level 1 (753.07 vs. 735.85 ms) (*z* = 0.22, *p* = 0.83).

#### Working Memory Demand Manipulation: Directly Comparing Experiments 1 and 2

##### Accuracy

There was a significant main effect of Memory Demand [*F*(1,215) = 4.68, *p* = 0.032], however there was no interaction between Age and Memory Demand [*F*(1,215) = 0.47, *p* = 0.49] or between Memory Demand and Task Switching [*F*(1,215) = 0.38, *p* = 0.54], suggesting that age group performance differences and switch costs were similar across both experiments. The main effect of Memory Demand was qualified by an interaction with Response Choice Number [*F*(1,214.99) = 61.09, *p* < 0.0001]. Overall, the absolute accuracy difference between four- and two- response choices was larger for high memory demand than low memory demand [9.96%, 95% CI (6.6,13.4), *z* = 5.76, *p* < 0.0001]. If we examine this effect by age, the difference between high and low demand for the four-choice levels was similar for adults [9.48%, 95% CI (3.92,15.05), *z* = 3.33, *p* = 0.0008] and children [7.04%, 95% CI (1.45,12.64), *z* = 2.47, *p* = 0.014]. There were no significant memory demand differences for task accuracy in the two-choice levels for adults (0.47%, *z* = 0.29, *p* = 0.77) or children (2.19%, *z* = 1.31, *p* = 0.19), suggesting that the biggest accuracy differences between low and high working memory demand were during four response choice levels (compare dashed and solid lines in **Figure 2B**).

##### Response times

Comparing across experiments, there was a main effect of Memory Demand [*F*(1,214.98) = 117.12, *p* < 0.0001], and this was qualified by interaction with Number of Response Choices [*F*(1,215.05) = 13.40), *p* = 0.0003] and Age [*F*(1,215.07) = 5.84, *p* = 0.016; **Figure 2D**], but not Task Switching [*F*(1,405.31) = 0.20, *p* = 0.66)]. The difference between low and high memory demand was greater in the four-choice levels than the two-choice levels (46.9 ms, 95% CI (21.7,72.2), *z* = 3.64, *p* = 0.0003). Moreover, the response time difference between low and high memory demand was greater in children (169.8 ms) relative to adults (91.3 ms) [diff = 78.4 ms, 95% CI (24.6, 132.3), *z* = 2.85, *p* = 0.004]. Overall, the difference in response time observed across low and high memory demand experiments was greater in the four choice levels than the two choice levels and greater in children than adults.

#### On-Task Learning within the Testing Period

When comparing within-session improvement in accuracy between both studies, there was an interaction between Level and Memory Demand [*F*(1,214.40) = 7.86, *p* = 0.006], but no significant three-way interaction with Age [*F*(1,214.40) = 1.28, *p* = 0.258]. There was an accuracy difference between high and low working memory demand in level 10 [4.24%, 95% CI (0.33,8.14), *z* = 2.13, *p* = 0.033], whereas level 1 did not show a baseline performance difference between high and low memory demand [1.82%, 95% CI (–2.07,5.73), *z* = 0.91, *p* = 0.36].

When comparing response time improvements across memory demand, there was a significant interaction between Level and Memory Demand [*F*(1,214.85) = 14.51, *p* = 0.0002], but no significant three-way interaction with Age [*F*(1,214.85) = 0.364, *p* = 0.55]. The relative improvement from level 1 to level 10 in response time was greater in the high memory demand condition than the low demand condition [55 ms, 95% CI (8,102), *z* = 2.28, *p* = 0.02]. If we examine this difference by age, the high memory demand condition showed a larger response time improvement by level 10 for adults [115 ms, 95% CI (80,150), *z* = 6.44, *p* < 0.0001] than in the low demand condition (60 ms, 95% (29, 92), *z* = 3.78, *p* = 0.0002]. The same larger improvement across levels occurred for children in the high memory demand condition [152 ms, 95% CI (116,187), *z* = 8.40, *p* < 0.0001] than the lower memory demand condition [76 ms, 95% CI (45,108), *z* = 8.4, *p* < 0.0001).

## Discussion

We manipulated numerous aspects of a cued switching game to compare the stability of task-switching abilities across children and adults. Our goals were (1) to attempt to narrow the gap between child and adult task-switching performance by either boosting child performance or degrading adult performance; (2) to study age-related performance differences more specifically (i.e., in smaller age bins) within a large cohort of children; and 3) to assess within-session improvement over age. Further, we measured the impact of two different amounts of working memory demand and three proportions of congruent stimuli on all of these outcomes (see **Tables 3–5**).

We found that both child and adult task performance were considerably robust to most task manipulations, showing strong changes only under the most difficult circumstances (four-feature, four-button switching) and under high working memory demand. For most levels, child performance appeared to become similar to that of adults in mid-adolescence (∼13 years) but accuracy differences remained for some challenging task manipulations. Importantly, increasing working memory demand and number of response choices increased variability in our child participants, resulting in different timing of this maturation across levels, suggesting that working memory capacity plays a significant developmental role in task-switching ability. Further, we saw earlier maturity of response times than accuracy, suggesting a future direction to study speed-accuracy trade-offs and individual variability in more detail in the performance transition ages of 12–16 year-olds (Lange-Küttner, 2012).

We found that both adult and child groups significantly improved with practice on the task, so that the last level (level 10), which was formatted the same as the first level, exhibited significant improvement in both response times and accuracy. This relative improvement after increasingly difficult task demands was significantly larger in the higher working memory load experiment. These results have important implications for reducing performance confounds in future behavioral and neuroimaging studies and are discussed in more detail below.

### Adults Outperformed Children Across Most Manipulations

Young adults outperformed children even at the easiest level (level 1) of both experiments, which was consistent with previous research (Crone et al., 2006b,c; Chevalier et al., 2013; Diamond, 2013; Holt and Deák, 2015; Church et al., 2017). This performance difference persisted despite removing lexical aspects of the task and substantially reducing working memory burden by keeping response choices on screen (Experiment 1). Thus, our attempts to narrow the gap in task-switching performance between children and adults were largely unsuccessful. In both experiments, children were consistently slower and less accurate than adults, and it proved challenging to degrade adult performance. The accuracy difference between groups widened only at the most challenging levels; adults were much less affected than children after we increased the number of potential responses from two to four. This differing impact on performance is consistent with previous research, which has found children are more impacted than adults by the presence of distractors in other domains like visual search (e.g., Enns and Cameron, 1987; Lange-Küttner and Bosco, 2016).

However, there was an unexpected and strikingly parallel response time pattern between groups observed in both Experiments 1 and 2, suggesting an age-related cost function in response times that is steady across our task manipulations. Adult performance only degraded under the hardest conditions: four features switching with four button choices, and high working memory load, where the target is alone on the screen (Experiment 2, levels 7–9).

Unexpectedly, our congruency manipulation had no significant effect on performance in either children or adults. We had predicted that children would receive an overall performance boost when a target was identical to one response choice, as these trials required no knowledge of the cued feature. While congruency needs to be explored further through a within-participant design, making up to 40% of the trials congruent did not significantly alter the performance gaps observed between child and adult groups.

Task switch costs in these experiments also did not differ between our child and adult samples. Thus, while overall performance showed a substantial improvement over age, switching on a trial-by-trial level was stable over age through a variety of task manipulations, including differing memory demand. Stable and consistent switch costs over age were also found in a visual recognition task (Lange-Küttner and Küttner, 2015). This result is particularly interesting when coupled with the observation of a steady trial response time cost and significant overall accuracy difference between age groups, and is worthy of more investigation.

The robustness of young adult cued switching performance in this set of experiments was remarkable. Of course, there are many types of task manipulations that we did not test here, a primary one being variation of the cue-target interval. We assume that shortening the large preparatory cue period (2000 ms) would impact performance at all ages by reducing advanced processing time (Meiran, 2000). Indeed, processing speed is a substantial factor in executive functioning, and substantially improves with age (Cepeda et al., 2013). Aiding child performance and encouraging preparatory processing were our motivations for a long cue period, in addition to a fMRI-friendly design that can separate cue and target processing (Ollinger et al., 2001).

### Critical Times of Change for Task-Switching Performance

There were clear developmental transitions in accuracy in our sample of children at ages 13 and above in Experiment 1, mostly within the two-button levels rather than the four-button levels. In Experiment 2, developmental transitions in terms of mean performance occurred mostly in many, but not all, of the two button levels. In the four button levels, while performance for the 13–16 year olds was not significantly different from adults, this was due to increased variability in performance overall. The 13–16 year old group means were qualitatively lower, congruent with Experiment 1. Better accuracy starting around adolescence was particularly clear in the more difficult levels of Experiment 1. Participants 13 years and older displayed a more consistent accuracy profile across the entire experiment, highly similar to what we observed in young adults.

This performance shift may be explained in part by previous findings demonstrating a task-dependent transition to greater proactive, or preparatory, cognitive control in adolescence (Braver, 2012; Munakata et al., 2012; Blackwell and Munakata, 2013). Increasing memory capacity, and thus the ability to maintain goal-directed behaviors, may underlie the greater preparatory engagement during the cue period in older children (Ghetti and Bunge, 2012). Our earlier fMRI work found that children in the same age range as this sample demonstrated less blood oxygenation level-dependent (BOLD) activity during the cue period of a cue-target trial than adults, and that this also improved around adolescence (Church et al., 2017). We interpreted the decreased cue activity in children as indicative of less preparatory cue processing. Here, we tested whether the lexical cues and high working memory demand used by Church et al. (2017) particularly hindered children from engaging adequate trial preparation. Experiments 1 and 2 used simple picture cues, and Experiment 1 had very low working memory demand relative to Church et al. (2017), and yet the performance gap between adults and children remained, supporting different preparatory cue processing over age as an explanation for both datasets.

It is also possible that the poorer performance observed in middle childhood groups both here and by Church et al. (2017) reflects difficulties in childhood with cue decoding, as the critical part of preparatory cue processing. Symbolic representation and the ability to mentally manipulate information are key parts of working memory development. Thus, the development of greater preparatory task control around age 12 years aligns with the behavioral results of our samples and others of working memory development and greater abstract thought processing (Cepeda et al., 2001; Crone et al., 2006b,c).

Another potential explanation for performance transitions in adolescence is a model of motivational cognitive flexibility in children undergoing pubertal changes proposed by Crone and Dahl (2012). They discuss how the combination of hormonal, neurological, and social changes during early adolescence supports the development of the ability to flexibly adjust goals and behavior in specific environmental contexts. This model of motivational cognitive flexibility aligns with the greater within-session improvements seen in the more challenging and engaging task design in the current study (Experiment 2). Examination of pubertal measures during this performance transition could provide greater information than chronological age about the timing and sources underlying task-switching improvement as measured here.

### Practice Facilitated Greater Improvement with Higher Memory Demand

The increased working memory demands in Experiment 2 promoted significantly more within-session improvement between levels 1 and 10 relative to Experiment 1, especially in children. This was true despite higher working memory demand in Experiment 2 leading to poor performance in children at the more difficult levels (i.e., levels 7, 8, and 9). The improvement observed in the high demand task may be driven by added engagement and challenge relative to the easier task (Locke and Braver, 2008; Crone and Dahl, 2012; Capa et al., 2013). In both experiments, child response times for level 10 became comparable to adult response times for level 1, though the group accuracy gap remained wide, especially with low memory demand. Interestingly, in a visual recognition study, the opposite pattern was found: child practiced accuracy grew more similar to adult initial accuracy, while response times remained different (Lange-Küttner, 2013). Our finding of response time overlap between level 1 and 10 may allow interesting group comparisons using fMRI, as a way to control for time on trial differences that are found when comparing age groups within level. It would be interesting to find if these behavioral improvements transfer to other types of stimuli during task switching; while our level 10 stimuli were different in shapes and colors from level 1, they were of the same type, and thus we cannot comment on whether behavioral improvement would transfer to dissimilar stimuli (cf. Kail and Park, 1990; Stransky et al., 2010).

The results here demonstrate that the processes necessary for successful use of preparatory task control (presumably working memory, sustained attention, and cognitive flexibility) can be improved upon with practice within a session in both age groups; however, we make no claims that these improvements were sustained over time (Klingberg, 2010; Capa et al., 2013). The significant improvements in the higher memory demand experiment suggest that manipulating working memory demand holds great promise as a means by which to improve task-switching ability in both children and adults within the context of a single session.

### Limitations

These experiments initially served as pilot studies for future neuroimaging work; as such, we did not collect neuropsychological assessments, apart from the task performance, that would better characterize our samples. However, our samples were carefully screened for any clinical diagnoses or medications, and were not receiving any special education services at school.

Experiment 2 was designed to increase working memory and cognitive load demands relative to Experiment 1, but it also differed in the amount of visual information available onscreen during the target period, thus potentially confounding memory demand with visual effects. A parallel analysis of the stimulus properties of the data (Martinez et al., 2017) indicates that the continued presence of the response choice information on the screen in Experiment 1 increases the likelihood that participants are swayed by visual stimulus similarity with response choices, and may not have encouraged loading of cue information in advance of the target. Future studies could attempt to separate out these visual crutches from memory demand.

## Conclusion

Our study of cued switching found consistent performance differences between children and adults, irrespective of working memory demand and number of response choices. We found higher working memory demand resulted in relatively steeper performance decrement at the highest levels, but, on the whole, children showed a consistent and flat response time cost relative to adults. A developmental shift in children’s performance to adult-like levels for both response times and accuracy occurred around early adolescence in both experiments. Sustained practice, through harder level exposure within session, pushed ‘experienced’ children to the response times of novice adults, and greater gains were seen in both groups with greater working memory demand. These results generate interesting future questions to study in children with control-related disorders, and in the overlap between learning and age in brain and behavior.

## Ethics Statement

This study was carried out in accordance with the Declaration of Helsinki, and the University of Texas Office of Research Support and Compliance, with written informed consent from all parents and adult participants, and written informed assent from all child participants. The protocol was approved by the University of Texas at Austin Institutional Review Board.

## Author Contributions

J–RB, JM, and MR collected the data. JC designed the experiment. J–RB, JM, and JC analyzed the data. J–RB, JM, and MR generated graphs of the data. JC, JM, J–RB wrote the paper. All authors edited the paper.

## Conflict of Interest Statement

The authors declare that the research was conducted in the absence of any commercial or financial relationships that could be construed as a potential conflict of interest.

## Footnotes

1 https://stat.ethz.ch/pipermail/r-help/2006-May/094765.html

2 http://glmm.wikidot.com/faq

3 https://osf.io/dmsy6/?view_only=524a052d3b684b41b8b863d185405adb

## Appendix

### Verbal Instructions for the Experiments

#### Practice Level:

“First I’m going to run the practice game in order to show you how to play. This is a rule-based matching game. There are three rows: the rule-row, the response choices row, and the target row (point to each row). Each time a target appears on the screen you choose the shape in the choices-row that matches it based on the rule, which is indicated by the red outline in the rule-row. As you can see, there are four possible rules you can follow: shape, inner color, pattern, and outer color. During the practice round you have unlimited time to choose the answer, but in the real game you will have 2 s to make your choice.
Right now, the rule is shape <press spacebar to bring up target> and the target is a red square. Which of these two response choices (point) do you think matches the target? (If they answer the circle, explain why the green square is the correct choice).
You will respond by using this controller. Hold the wire on the right side and use your thumbs to press the buttons. Right now, there are only two cues, so you only use the inner two buttons. Later on, there will be four response choices, and you will use all buttons as they correspond to the position of the choices on the screen. In some of the levels, the choices will change, in others they will stay the same. Do you have any questions?”

Before each of the levels say something similar to the following:

Level 1: “This is the first level of the game. It will be similar to the practice you just did. The only rules are shape and inner color and only two buttons. You have 2 s to respond this time, so if you miss one, just try and respond to the next one. Do the best you can.”
Level 2: “This level will be similar to the first one.”
Level 3: “We are now adding the third rule, which is pattern. You’re going to start seeing some patterns in the response choices. If the pattern rule pops up then just match based on the pattern.”
Level 4: “We are adding the last rule now: outer color. The border of the choices will now be filled in, if the outer color rule pops up then match based on the outside border color.”
Level 5: “Nothing new, all four rules are still active. After this you are half way done.”
Level 6: “This will be the last two button level. After this we will take off the button caps and you’ll play with four answer choices”
Level 7: **Take off button caps** – “For the next three levels, there will now be four response choices on the screen to remember and choose from.”
Level 8: “Almost done!”
Level 9: “Last four button level. After this is the last level which will be the same as the first one you did to see how you have improved.”
Level 10: **Put button caps back on** “This is the last level. You will only see the shape and inner color rule and only two response choices.”
